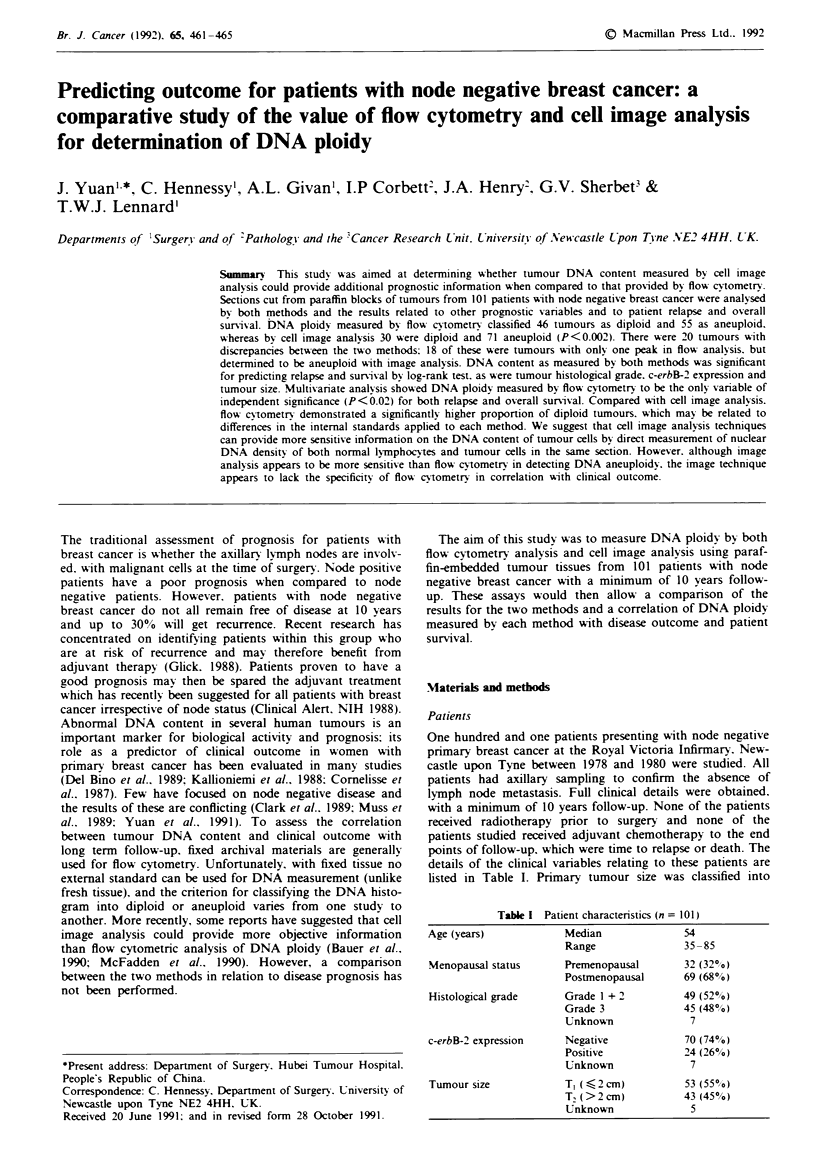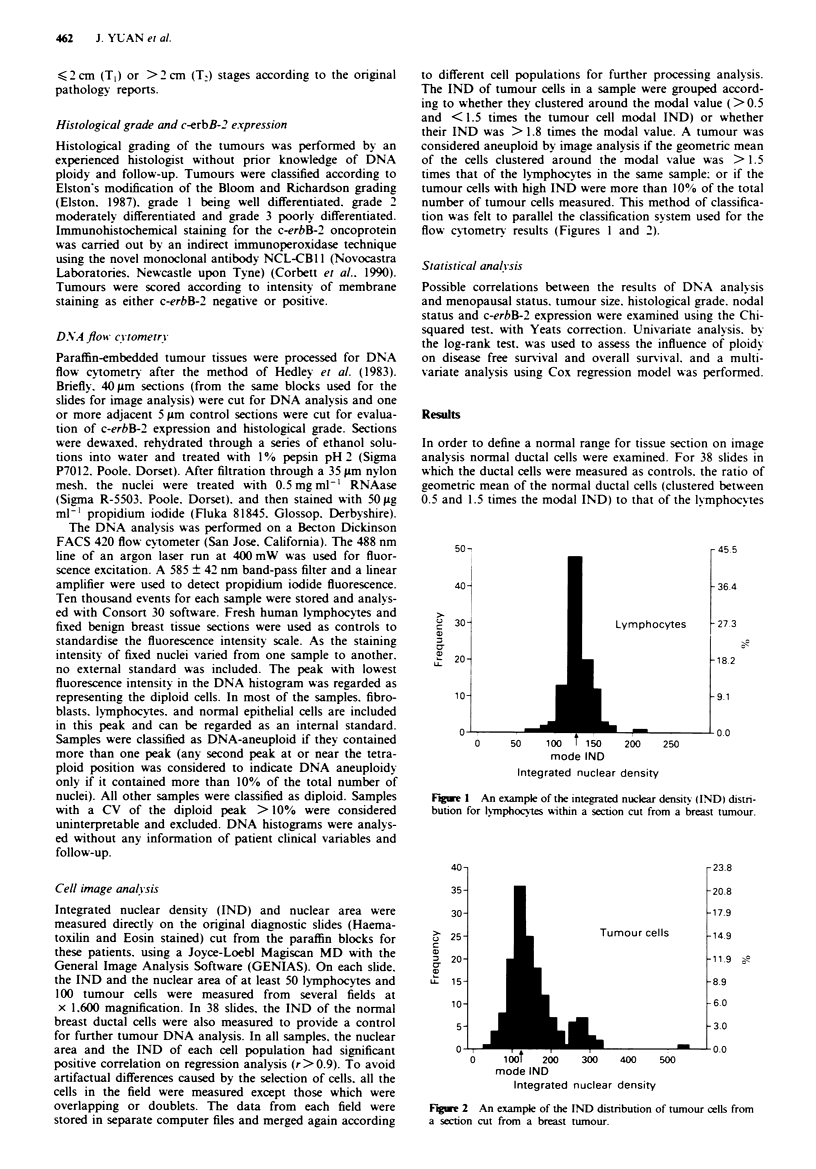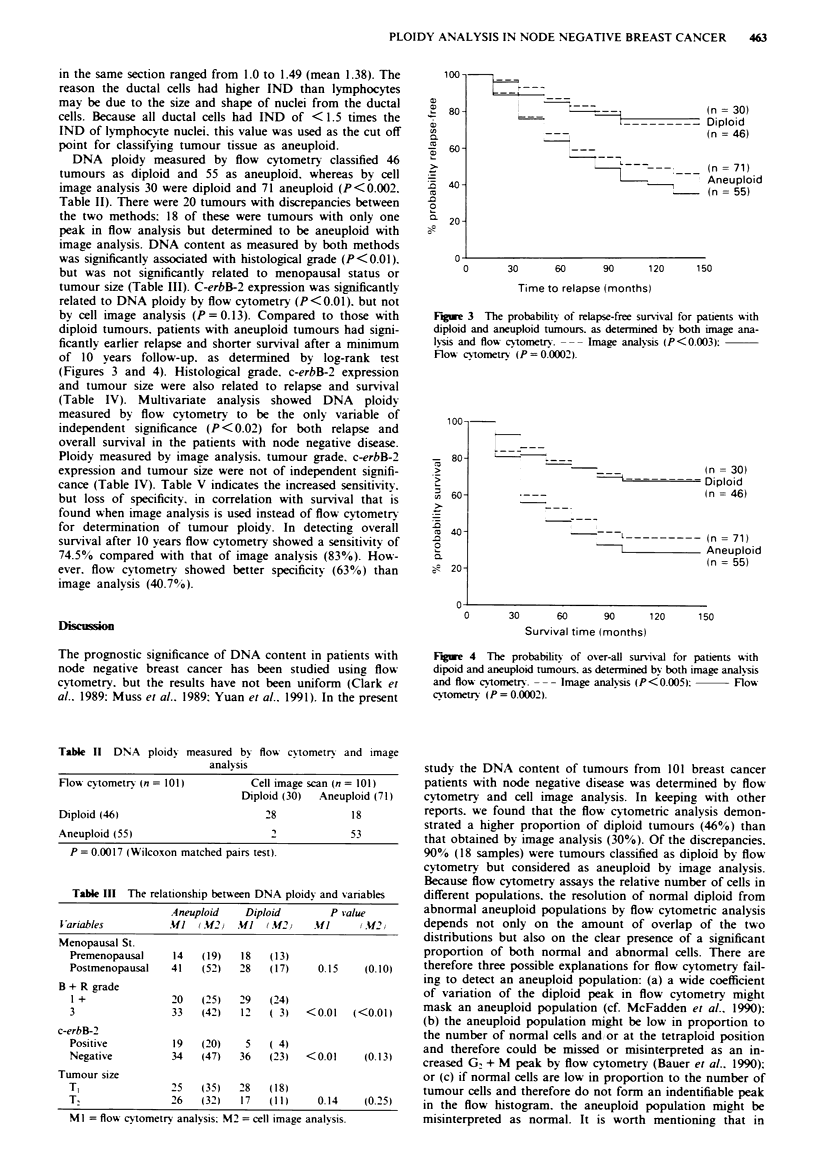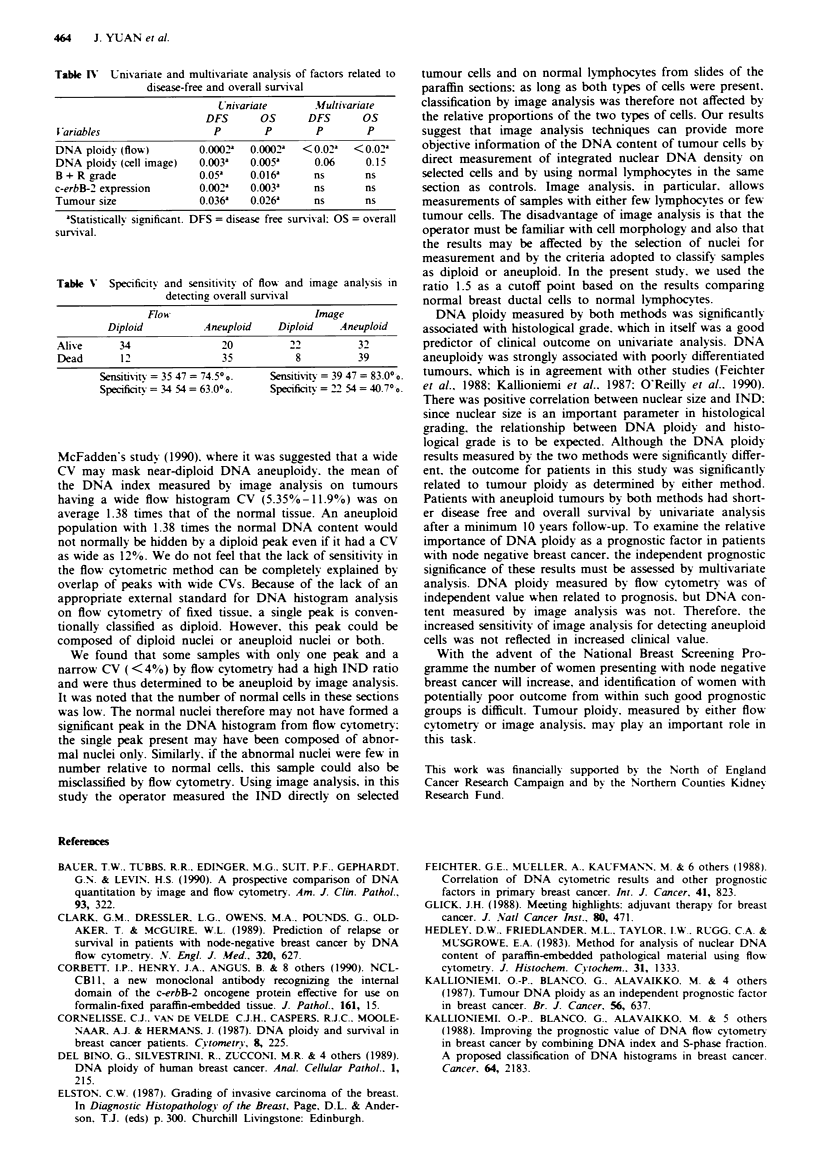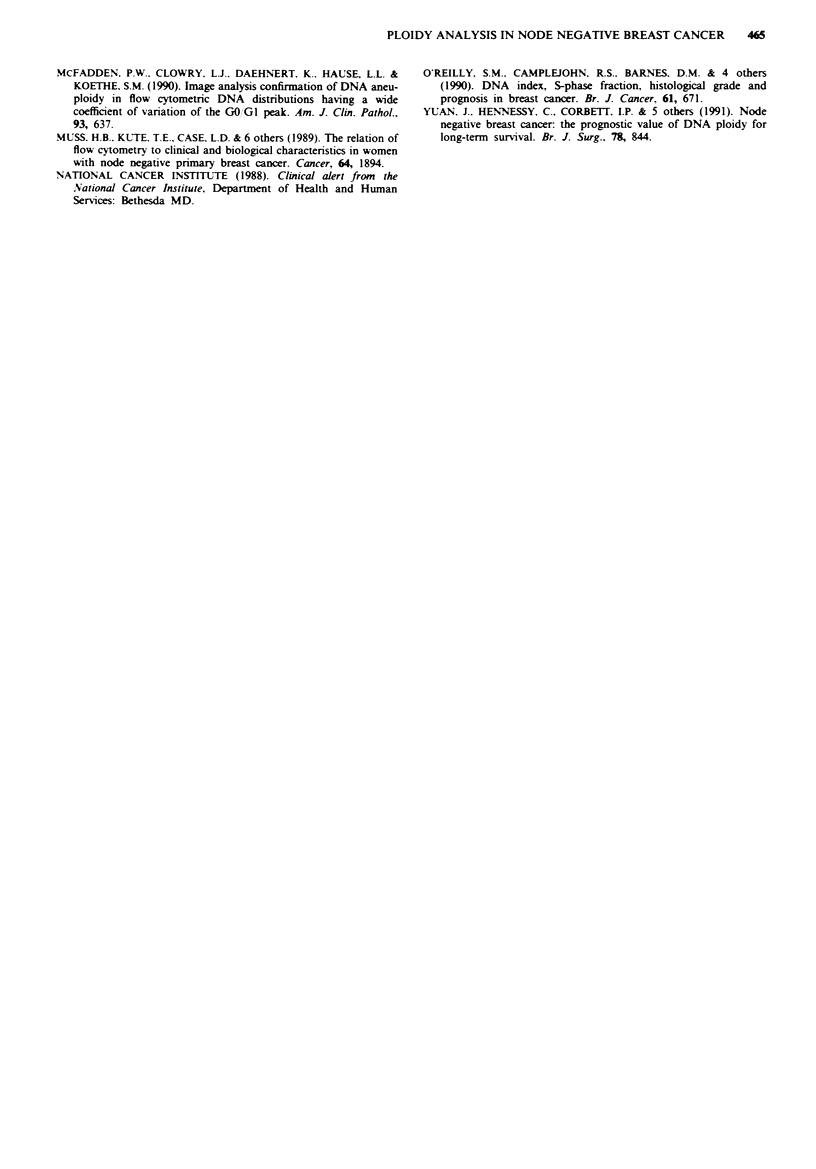# Predicting outcome for patients with node negative breast cancer: a comparative study of the value of flow cytometry and cell image analysis for determination of DNA ploidy.

**DOI:** 10.1038/bjc.1992.93

**Published:** 1992-03

**Authors:** J. Yuan, C. Hennessy, A. L. Givan, I. P. Corbett, J. A. Henry, G. V. Sherbet, T. W. Lennard

**Affiliations:** Department of Surgery, University of Newcastle Upon Tyne, UK.

## Abstract

This study was aimed at determining whether tumour DNA content measured by cell image analysis could provide additional prognostic information when compared to that provided by flow cytometry. Sections cut from paraffin blocks of tumours from 101 patients with node negative breast cancer were analysed by both methods and the results related to other prognostic variables and to patient relapse and overall survival. DNA ploidy measured by flow cytometry classified 46 tumours as diploid and 55 as aneuploid, whereas by cell image analysis 30 were diploid and 71 aneuploid (P less than 0.002). There were 20 tumours with discrepancies between the two methods; 18 of these were tumours with only one peak in flow analysis, but determined to be aneuploid with image analysis. DNA content as measured by both methods was significant for predicting relapse and survival by log-rank test, as were tumour histological grade, c-erbB-2 expression and tumour size. Multivariate analysis showed DNA ploidy measured by flow cytometry to be the only variable of independent significance (P less than 0.02) for both relapse and overall survival. Compared with cell image analysis, flow cytometry demonstrated a significantly higher proportion of diploid tumours, which may be related to differences in the internal standards applied to each method. We suggest that cell image analysis techniques can provide more sensitive information on the DNA content of tumour cells by direct measurement of nuclear DNA density of both normal lymphocytes and tumour cells in the same section. However, although image analysis appears to be more sensitive than flow cytometry in detecting DNA aneuploidy, the image technique appears to lack the specificity of flow cytometry in correlation with clinical outcome.


Br. J. Cancer (1992), 65, 461-465 © Macmillan Press Ltd., 1992

### Predicting outcome for patients with node negative breast cancer: a comparative study of the value of flow cytometry and cell image analysis for determination of DNA ploidy

J. Yuan[1,*], C. Hennessy[1], A.L. Givan[1], I.P Corbett[2], J.A. Henry[2], G.V. Sherbet[3] & T.W.J. Lennard[1]

*Departments of [1]Surgery and of [2]Pathology and the [3]Cancer Research Unit, University of Newcastle Upon Tyne NE2 4HH, UK.*

**Summary** This study was aimed at determining whether tumour DNA content measured by cell image analysis could provide additional prognostic information when compared to that provided by flow cytometry. Sections cut from paraffin blocks of tumours from 101 patients with node negative breast cancer were analysed by both methods and the results related to other prognostic variables and to patient relapse and overall survival. DNA ploidy measured by flow cytometry classified 46 tumours as diploid and 55 as aneuploid, whereas by cell image analysis 30 were diploid and 71 aneuploid ($P<0.002$). There were 20 tumours with discrepancies between the two methods; 18 of these were tumours with only one peak in flow analysis, but determined to be aneuploid with image analysis. DNA content as measured by both methods was significant for predicting relapse and survival by log-rank test, as were tumour histological grade, c-*erb*B-2 expression and tumour size. Multivariate analysis showed DNA ploidy measured by flow cytometry to be the only variable of independent significance ($P<0.02$) for both relapse and overall survival. Compared with cell image analysis, flow cytometry demonstrated a significantly higher proportion of diploid tumours, which may be related to differences in the internal standards applied to each method. We suggest that cell image analysis techniques can provide more sensitive information on the DNA content of tumour cells by direct measurement of nuclear DNA density of both normal lymphocytes and tumour cells in the same section. However, although image analysis appears to be more sensitive than flow cytometry in detecting DNA aneuploidy, the image technique appears to lack the specificity of flow cytometry in correlation with clinical outcome.

The traditional assessment of prognosis for patients with breast cancer is whether the axillary lymph nodes are involved, with malignant cells at the time of surgery. Node positive patients have a poor prognosis when compared to node negative patients. However, patients with node negative breast cancer do not all remain free of disease at 10 years and up to 30% will get recurrence. Recent research has concentrated on identifying patients within this group who are at risk of recurrence and may therefore benefit from adjuvant therapy (Glick, 1988). Patients proven to have a good prognosis may then be spared the adjuvant treatment which has recently been suggested for all patients with breast cancer irrespective of node status (Clinical Alert, NIH 1988). Abnormal DNA content in several human tumours is an important marker for biological activity and prognosis; its role as a predictor of clinical outcome in women with primary breast cancer has been evaluated in many studies (Del Bino *et al*., 1989; Kallioniemi *et al*., 1988; Cornelisse *et al*., 1987). Few have focused on node negative disease and the results of these are conflicting (Clark *et al*., 1989; Muss *et al*., 1989; Yuan *et al*., 1991). To assess the correlation between tumour DNA content and clinical outcome with long term follow-up, fixed archival materials are generally used for flow cytometry. Unfortunately, with fixed tissue no external standard can be used for DNA measurement (unlike fresh tissue), and the criterion for classifying the DNA histogram into diploid or aneuploid varies from one study to another. More recently, some reports have suggested that cell image analysis could provide more objective information than flow cytometric analysis of DNA ploidy (Bauer *et al*., 1990; McFadden *et al*., 1990). However, a comparison between the two methods in relation to disease prognosis has not been performed.

*Present address: Department of Surgery, Hubei Tumour Hospital, People's Republic of China.
Correspondence: C. Hennessy, Department of Surgery, University of Newcastle upon Tyne NE2 4HH, UK.
Received 20 June 1991; and in revised form 28 October 1991.

The aim of this study was to measure DNA ploidy by both flow cytometry analysis and cell image analysis using paraffin-embedded tumour tissues from 101 patients with node negative breast cancer with a minimum of 10 years follow-up. These assays would then allow a comparison of the results for the two methods and a correlation of DNA ploidy measured by each method with disease outcome and patient survival.

### Materials and methods

### Patients

One hundred and one patients presenting with node negative primary breast cancer at the Royal Victoria Infirmary, Newcastle upon Tyne between 1978 and 1980 were studied. All patients had axillary sampling to confirm the absence of lymph node metastasis. Full clinical details were obtained, with a minimum of 10 years follow-up. None of the patients received radiotherapy prior to surgery and none of the patients studied received adjuvant chemotherapy to the end points of follow-up, which were time to relapse or death. The details of the clinical variables relating to these patients are listed in Table I. Primary tumour size was classified into

**Table I** Patient characteristics ($n$ = 101)

| | | |
|---|---|---|
| Age (years) | Median | 54 |
| | Range | 35-85 |
| Menopausal status | Premenopausal | 32 (32%) |
| | Postmenopausal | 69 (68%) |
| Histological grade | Grade 1 + 2 | 49 (52%) |
| | Grade 3 | 45 (48%) |
| | Unknown | 7 |
| c-*erb*B-2 expression | Negative | 70 (74%) |
| | Positive | 24 (26%) |
| | Unknown | 7 |
| Tumour size | $T_1$ ($\leq$2 cm) | 53 (55%) |
| | $T_2$ (>2 cm) | 43 (45%) |
| | Unknown | 5 |

$\leq$2 cm ($T_1$) or >2 cm ($T_2$) stages according to the original pathology reports.

*Histological grade and c-erbB-2 expression*

Histological grading of the tumours was performed by an experienced histologist without prior knowledge of DNA ploidy and follow-up. Tumours were classified according to Elston's modification of the Bloom and Richardson grading (Elston, 1987), grade 1 being well differentiated, grade 2 moderately differentiated and grade 3 poorly differentiated. Immunohistochemical staining for the c-*erb*B-2 oncoprotein was carried out by an indirect immunoperoxidase technique using the novel monoclonal antibody NCL-CB11 (Novocastra Laboratories, Newcastle upon Tyne) (Corbett *et al.*, 1990). Tumours were scored according to intensity of membrane staining as either c-*erb*B-2 negative or positive.

*DNA flow cytometry*

Paraffin-embedded tumour tissues were processed for DNA flow cytometry after the method of Hedley *et al.* (1983). Briefly, 40 μm sections (from the same blocks used for the slides for image analysis) were cut for DNA analysis and one or more adjacent 5 μm control sections were cut for evaluation of c-*erb*B-2 expression and histological grade. Sections were dewaxed, rehydrated through a series of ethanol solutions into water and treated with 1% pepsin pH 2 (Sigma P7012, Poole, Dorset). After filtration through a 35 μm nylon mesh, the nuclei were treated with 0.5 mg $ml^{-1}$ RNAase (Sigma R-5503, Poole, Dorset), and then stained with 50 μg $ml^{-1}$ propidium iodide (Fluka 81845, Glossop, Derbyshire).

The DNA analysis was performed on a Becton Dickinson FACS 420 flow cytometer (San Jose, California). The 488 nm line of an argon laser run at 400 mW was used for fluorscence excitation. A 585 ± 42 nm band-pass filter and a linear amplifier were used to detect propidium iodide fluorescence. Ten thousand events for each sample were stored and analysed with Consort 30 software. Fresh human lymphocytes and fixed benign breast tissue sections were used as controls to standardise the fluorescence intensity scale. As the staining intensity of fixed nuclei varied from one sample to another, no external standard was included. The peak with lowest fluorescence intensity in the DNA histogram was regarded as representing the diploid cells. In most of the samples, fibroblasts, lymphocytes, and normal epithelial cells are included in this peak and can be regarded as an internal standard. Samples were classified as DNA-aneuploid if they contained more than one peak (any second peak at or near the tetraploid position was considered to indicate DNA aneuploidy only if it contained more than 10% of the total number of nuclei). All other samples were classified as diploid. Samples with a CV of the diploid peak >10% were considered uninterpretable and excluded. DNA histograms were analysed without any information of patient clinical variables and follow-up.

*Cell image analysis*

Integrated nuclear density (IND) and nuclear area were measured directly on the original diagnostic slides (Haematoxilin and Eosin stained) cut from the paraffin blocks for these patients, using a Joyce-Loebl Magiscan MD with the General Image Analysis Software (GENIAS). On each slide, the IND and the nuclear area of at least 50 lymphocytes and 100 tumour cells were measured from several fields at × 1,600 magnification. In 38 slides, the IND of the normal breast ductal cells were also measured to provide a control for further tumour DNA analysis. In all samples, the nuclear area and the IND of each cell population had significant positive correlation on regression analysis ($r$>0.9). To avoid artifactual differences caused by the selection of cells, all the cells in the field were measured except those which were overlapping or doublets. The data from each field were stored in separate computer files and merged again according to different cell populations for further processing analysis. The IND of tumour cells in a sample were grouped according to whether they clustered around the modal value (>0.5 and <1.5 times the tumour cell modal IND) or whether their IND was >1.8 times the modal value. A tumour was considered aneuploid by image analysis if the geometric mean of the cells clustered around the modal value was >1.5 times that of the lymphocytes in the same sample; or if the tumour cells with high IND were more than 10% of the total number of tumour cells measured. This method of classification was felt to parallel the classification system used for the flow cytometry results (Figures 1 and 2).

*Statistical analysis*

Possible correlations between the results of DNA analysis and menopausal status, tumour size, histological grade, nodal status and c-*erb*B-2 expression were examined using the Chi-squared test, with Yeats correction. Univariate analysis, by the log-rank test, was used to assess the influence of ploidy on disease free survival and overall survival, and a multivariate analysis using Cox regression model was performed.

### Results

In order to define a normal range for tissue section on image analysis normal ductal cells were examined. For 38 slides in which the ductal cells were measured as controls, the ratio of geometric mean of the normal ductal cells (clustered between 0.5 and 1.5 times the modal IND) to that of the lymphocytes

**Figure 1** An example of the integrated nuclear density (IND) distribution for lymphocytes within a section cut from a breast tumour.

**Figure 2** An example of the IND distribution of tumour cells from a section cut from a breast tumour.

in the same section ranged from 1.0 to 1.49 (mean 1.38). The reason the ductal cells had higher IND than lymphocytes may be due to the size and shape of nuclei from the ductal cells. Because all ductal cells had IND of $<1.5$ times the IND of lymphocyte nuclei, this value was used as the cut off point for classifying tumour tissue as aneuploid.

DNA ploidy measured by flow cytometry classified 46 tumours as diploid and 55 as aneuploid, whereas by cell image analysis 30 were diploid and 71 aneuploid ($P<0.002$, Table II). There were 20 tumours with discrepancies between the two methods; 18 of these were tumours with only one peak in flow analysis but determined to be aneuploid with image analysis. DNA content as measured by both methods was significantly associated with histological grade ($P<0.01$), but was not significantly related to menopausal status or tumour size (Table III). C-*erb*B-2 expression was significantly related to DNA ploidy by flow cytometry ($P<0.01$), but not by cell image analysis ($P=0.13$). Compared to those with diploid tumours, patients with aneuploid tumours had significantly earlier relapse and shorter survival after a minimum of 10 years follow-up, as determined by log-rank test (Figures 3 and 4). Histological grade, c-*erb*B-2 expression and tumour size were also related to relapse and survival (Table IV). Multivariate analysis showed DNA ploidy measured by flow cytometry to be the only variable of independent significance ($P<0.02$) for both relapse and overall survival in the patients with node negative disease. Ploidy measured by image analysis, tumour grade, c-*erb*B-2 expression and tumour size were not of independent significance (Table IV). Table V indicates the increased sensitivity, but loss of specificity, in correlation with survival that is found when image analysis is used instead of flow cytometry for determination of tumour ploidy. In detecting overall survival after 10 years flow cytometry showed a sensitivity of 74.5% compared with that of image analysis (83%). However, flow cytometry showed better specificity (63%) than image analysis (40.7%).

**Figure 3** The probability of relapse-free survival for patients with diploid and aneuploid tumours, as determined by both image analysis and flow cytometry. --- Image analysis ($P<0.003$); ——— Flow cytometry ($P=0.0002$).

**Figure 4** The probability of over-all survival for patients with dipoid and aneuploid tumours, as determined by both image analysis and flow cytometry. --- Image analysis ($P<0.005$); ——— Flow cytometry ($P=0.0002$).

### Discussion

The prognostic significance of DNA content in patients with node negative breast cancer has been studied using flow cytometry, but the results have not been uniform (Clark *et al.*, 1989; Muss *et al.*, 1989; Yuan *et al.*, 1991). In the present study the DNA content of tumours from 101 breast cancer patients with node negative disease was determined by flow cytometry and cell image analysis. In keeping with other reports, we found that the flow cytometric analysis demonstrated a higher proportion of diploid tumours (46%) than that obtained by image analysis (30%). Of the discrepancies, 90% (18 samples) were tumours classified as diploid by flow cytometry but considered as aneuploid by image analysis. Because flow cytometry assays the relative number of cells in different populations, the resolution of normal diploid from abnormal aneuploid populations by flow cytometric analysis depends not only on the amount of overlap of the two distributions but also on the clear presence of a significant proportion of both normal and abnormal cells. There are therefore three possible explanations for flow cytometry failing to detect an aneuploid population: (a) a wide coefficient of variation of the diploid peak in flow cytometry might mask an aneuploid population (cf. McFadden *et al.*, 1990); (b) the aneuploid population might be low in proportion to the number of normal cells and/or at the tetraploid position and therefore could be missed or misinterpreted as an increased $G_2 + M$ peak by flow cytometry (Bauer *et al.*, 1990); or (c) if normal cells are low in proportion to the number of tumour cells and therefore do not form an indentifiable peak in the flow histogram, the aneuploid population might be misinterpreted as normal. It is worth mentioning that in

**Table II** DNA ploidy measured by flow cytometry and image analysis

| Flow cytometry ($n = 101$) | Cell image scan ($n = 101$) Diploid (30) | Aneuploid (71) |
|---|---|---|
| Diploid (46) | 28 | 18 |
| Aneuploid (55) | 2 | 53 |

$P = 0.0017$ (Wilcoxon matched pairs test).

**Table III** The relationship between DNA ploidy and variables

| *Variables* | *Aneuploid* *M1* | *(M2)* | *Diploid* *M1* | *(M2)* | *P value* *M1* | *(M2)* |
|---|---|---|---|---|---|---|
| Menopausal St. | | | | | | |
| Premenopausal | 14 | (19) | 18 | (13) | | |
| Postmenopausal | 41 | (52) | 28 | (17) | 0.15 | (0.10) |
| B + R grade | | | | | | |
| 1 + | 20 | (25) | 29 | (24) | | |
| 3 | 33 | (42) | 12 | ( 3) | <0.01 | (<0.01) |
| c-*erb*B-2 | | | | | | |
| Positive | 19 | (20) | 5 | ( 4) | | |
| Negative | 34 | (47) | 36 | (23) | <0.01 | (0.13) |
| Tumour size | | | | | | |
| $T_1$ | 25 | (35) | 28 | (18) | | |
| $T_2$ | 26 | (32) | 17 | (11) | 0.14 | (0.25) |

M1 = flow cytometry analysis; M2 = cell image analysis.

**Table IV** Univariate and multivariate analysis of factors related to disease-free and overall survival

| Variables | Univariate DFS P | Univariate OS P | Multivariate DFS P | Multivariate OS P |
|---|---|---|---|---|
| DNA ploidy (flow) | 0.0002[a] | 0.0002[a] | <0.02[a] | <0.02[a] |
| DNA ploidy (cell image) | 0.003[a] | 0.005[a] | 0.06 | 0.15 |
| B + R grade | 0.05[a] | 0.016[a] | ns | ns |
| c-*erb*B-2 expression | 0.002[a] | 0.003[a] | ns | ns |
| Tumour size | 0.036[a] | 0.026[a] | ns | ns |

[a]Statistically significant. DFS = disease free survival; OS = overall survival.

**Table V** Specificity and sensitivity of flow and image analysis in detecting overall survival

| | Flow Diploid | Flow Aneuploid | Image Diploid | Image Aneuploid |
|---|---|---|---|---|
| Alive | 34 | 20 | 22 | 32 |
| Dead | 12 | 35 | 8 | 39 |

Sensitivity = 35 47 = 74.5%. Specificity = 34 54 = 63.0%.

Sensitivity = 39 47 = 83.0%. Specificity = 22 54 = 40.7%.

McFadden's study (1990), where it was suggested that a wide CV may mask near-diploid DNA aneuploidy, the mean of the DNA index measured by image analysis on tumours having a wide flow histogram CV (5.35%–11.9%) was on average 1.38 times that of the normal tissue. An aneuploid population with 1.38 times the normal DNA content would not normally be hidden by a diploid peak even if it had a CV as wide as 12%. We do not feel that the lack of sensitivity in the flow cytometric method can be completely explained by overlap of peaks with wide CVs. Because of the lack of an appropriate external standard for DNA histogram analysis on flow cytometry of fixed tissue, a single peak is conventionally classified as diploid. However, this peak could be composed of diploid nuclei or aneuploid nuclei or both.

We found that some samples with only one peak and a narrow CV (<4%) by flow cytometry had a high IND ratio and were thus determined to be aneuploid by image analysis. It was noted that the number of normal cells in these sections was low. The normal nuclei therefore may not have formed a significant peak in the DNA histogram from flow cytometry: the single peak present may have been composed of abnormal nuclei only. Similarly, if the abnormal nuclei were few in number relative to normal cells, this sample could also be misclassified by flow cytometry. Using image analysis, in this study the operator measured the IND directly on selected tumour cells and on normal lymphocytes from slides of the paraffin sections; as long as both types of cells were present, classification by image analysis was therefore not affected by the relative proportions of the two types of cells. Our results suggest that image analysis techniques can provide more objective information of the DNA content of tumour cells by direct measurement of integrated nuclear DNA density on selected cells and by using normal lymphocytes in the same section as controls. Image analysis, in particular, allows measurements of samples with either few lymphocytes or few tumour cells. The disadvantage of image analysis is that the operator must be familiar with cell morphology and also that the results may be affected by the selection of nuclei for measurement and by the criteria adopted to classify samples as diploid or aneuploid. In the present study, we used the ratio 1.5 as a cutoff point based on the results comparing normal breast ductal cells to normal lymphocytes.

DNA ploidy measured by both methods was significantly associated with histological grade, which in itself was a good predictor of clinical outcome on univariate analysis. DNA aneuploidy was strongly associated with poorly differentiated tumours, which is in agreement with other studies (Feichter *et al.*, 1988; Kallioniemi *et al.*, 1987; O'Reilly *et al.*, 1990). There was positive correlation between nuclear size and IND; since nuclear size is an important parameter in histological grading, the relationship between DNA ploidy and histological grade is to be expected. Although the DNA ploidy results measured by the two methods were significantly different, the outcome for patients in this study was significantly related to tumour ploidy as determined by either method. Patients with aneuploid tumours by both methods had shorter disease free and overall survival by univariate analysis after a minimum 10 years follow-up. To examine the relative importance of DNA ploidy as a prognostic factor in patients with node negative breast cancer, the independent prognostic significance of these results must be assessed by multivariate analysis. DNA ploidy measured by flow cytometry was of independent value when related to prognosis, but DNA content measured by image analysis was not. Therefore, the increased sensitivity of image analysis for detecting aneuploid cells was not reflected in increased clinical value.

With the advent of the National Breast Screening Programme the number of women presenting with node negative breast cancer will increase, and identification of women with potentially poor outcome from within such good prognostic groups is difficult. Tumour ploidy, measured by either flow cytometry or image analysis, may play an important role in this task.

This work was financially supported by the North of England Cancer Research Campaign and by the Northern Counties Kidney Research Fund.

### References

BAUER, T.W., TUBBS, R.R., EDINGER, M.G., SUIT, P.F., GEPHARDT, G.N. & LEVIN, H.S. (1990). A prospective comparison of DNA quantitation by image and flow cytometry. *Am. J. Clin. Pathol.*, **93**, 322.

CLARK, G.M., DRESSLER, L.G., OWENS, M.A., POUNDS, G., OLDAKER, T. & MCGUIRE, W.L. (1989). Prediction of relapse or survival in patients with node-negative breast cancer by DNA flow cytometry. *N. Engl. J. Med.*, **320**, 627.

CORBETT, I.P., HENRY, J.A., ANGUS, B. & 8 others (1990). NCL-CB11, a new monoclonal antibody recognizing the internal domain of the c-*erb*B-2 oncogene protein effective for use on formalin-fixed paraffin-embedded tissue. *J. Pathol.*, **161**, 15.

CORNELISSE, C.J., VAN DE VELDE C.J.H., CASPERS, R.J.C., MOOLENAAR, A.J. & HERMANS, J. (1987). DNA ploidy and survival in breast cancer patients. *Cytometry*, **8**, 225.

DEL BINO, G., SILVESTRINI, R., ZUCCONI, M.R. & 4 others (1989). DNA ploidy of human breast cancer. *Anal. Cellular Pathol.*, **1**, 215.

ELSTON, C.W. (1987). Grading of invasive carcinoma of the breast. In *Diagnostic Histopathology of the Breast*, Page, D.L. & Anderson, T.J. (eds) p. 300. Churchill Livingstone: Edinburgh.

FEICHTER, G.E., MUELLER, A., KAUFMANN, M. & 6 others (1988). Correlation of DNA cytometric results and other prognostic factors in primary breast cancer. *Int. J. Cancer*, **41**, 823.

GLICK, J.H. (1988). Meeting highlights: adjuvant therapy for breast cancer. *J. Natl Cancer Inst.*, **80**, 471.

HEDLEY, D.W., FRIEDLANDER, M.L., TAYLOR, I.W., RUGG, C.A. & MUSGROWE, E.A. (1983). Method for analysis of nuclear DNA content of paraffin-embedded pathological material using flow cytometry. *J. Histochem. Cytochem.*, **31**, 1333.

KALLIONIEMI, O.-P., BLANCO, G., ALAVAIKKO, M. & 4 others (1987). Tumour DNA ploidy as an independent prognostic factor in breast cancer. *Br. J. Cancer*, **56**, 637.

KALLIONIEMI, O.-P., BLANCO, G., ALAVAIKKO, M. & 5 others (1988). Improving the prognostic value of DNA flow cytometry in breast cancer by combining DNA index and S-phase fraction. A proposed classification of DNA histograms in breast cancer. *Cancer*, **64**, 2183.

MCFADDEN, P.W., CLOWRY, L.J., DAEHNERT, K., HAUSE, L.L. & KOETHE, S.M. (1990). Image analysis confirmation of DNA aneuploidy in flow cytometric DNA distributions having a wide coefficient of variation of the G0/G1 peak. *Am. J. Clin. Pathol.*, **93**, 637.
MUSS, H.B., KUTE, T.E., CASE, L.D. & 6 others (1989). The relation of flow cytometry to clinical and biological characteristics in women with node negative primary breast cancer. *Cancer*, **64**, 1894.
NATIONAL CANCER INSTITUTE (1988). *Clinical alert from the National Cancer Institute*, Department of Health and Human Services: Bethesda MD.
O'REILLY, S.M., CAMPLEJOHN, R.S., BARNES, D.M. & 4 others (1990). DNA index, S-phase fraction, histological grade and prognosis in breast cancer. *Br. J. Cancer*, **61**, 671.
YUAN, J., HENNESSY, C., CORBETT, I.P. & 5 others (1991). Node negative breast cancer: the prognostic value of DNA ploidy for long-term survival. *Br. J. Surg.*, **78**, 844.